# Relative peripheral refraction across 4 meridians after orthokeratology and LASIK surgery

**DOI:** 10.1186/s40662-018-0106-1

**Published:** 2018-05-20

**Authors:** António Queirós, Ana Amorim-de-Sousa, Daniela Lopes-Ferreira, César Villa-Collar, Ángel Ramón Gutiérrez, José Manuel González-Méijome

**Affiliations:** 1 Clinical & Experimental Optometry Research Lab-CEORLab, Center of Physics (Optometry), University of Minho, Braga, Portugal; 2 Department of Optics and Optometry, European University of Madrid, Madrid, Spain; 3 Department of Ophthalmology, University of Murcia, Murcia, Spain

**Keywords:** LASIK surgery, Orthokeratology, Peripheral refraction, Myopia progression

## Abstract

**Background:**

To characterize the axial and off-axis refraction across four meridians of the retina in myopic eyes before and after Orthokeratology (OK) and LASIK surgery.

**Methods:**

Sixty right eyes with a spherical equivalent (M) between − 0.75 to − 5.25 D (cylinder <− 1.00 D) underwent LASIK (n = 26) or OK (n = 34) to treat myopia. Axial and off-axis refraction were measured with an open-field autorefractometer before and after stabilized treatments. Off-axis measurements were obtained for the horizontal (35° nasal and temporal retina) and vertical (15° superior and inferior retina) meridians, and for two oblique directions (45–225° and 135–315°) up to 20° of eccentricity. The refractive profile was addressed as relative peripheral refractive error (RPRE).

**Results:**

OK and LASIK post-treatment results showed an increase of myopic relative refraction at several eccentric locations. At the four meridians evaluated, the M component of the pre-treatment RPRE values was not statistically different (*p* > 0.05) from the post-treatment RPRE within 30° and 20° of the central visual field after LASIK and OK, respectively. These results demonstrated that the treatment zone warrants an optimal central field of vision.

**Conclusions:**

The present study gives an overview of RPRE after refractive corneal reshaping treatments (OK and LASIK) across vertical, horizontal and two oblique meridians together. This allows a 3D representation of RPRE at the retina and shows that the myopic shift induced by both treatments is more relevant in horizontal directions.

## Background

Myopia prevalence vary from 30% in America or Europe to 70% in East Asia [1–4]. Several risk factors for myopia have been identified in the last decade, and is now classified a public health concern [5, 6].

Current knowledge suggests that the progression of the refractive error is related to the peripheral refraction. This behavior is supported by the ability of the optical defocus in the peripheral retina to regulate ocular growth and the emmetropization process in animal models. However, this biological process is not fully understood [7].

There are optical treatments that change the hyperope peripheral refraction of myopic eyes to a myopic peripheral profile [8, 9]. These changes were well depicted over the horizontal visual field after orthokeratology (OK) by Queirós et al. [9] and Mathur et al. [10]. The paracentral myopization in OK treatment increases the optical power of the corneal area surrounding the treatment zone [11]. These corneal changes have been pointed to as the mechanism to slow down axial eye growth associated to this treatment in case reports [12] and in controlled clinical trials in different countries and ethnicities [13–16].

Over the last two decades, people with low-to-moderate refractive errors, particularly myopic patients, have preferred the corneal refractive surgery as a corrective option. Laser-Assisted in Situ Keratomileusis (LASIK) allows to reduce the dependence on spectacles or contact lenses. Although LASIK is not applied with the purpose for regulating myopia, the peripheral refraction and peripheral image quality has been previously characterized along the horizontal meridian [17–20].

The possible role of the peripheral refractive profile over the horizontal meridian in the onset and progression of myopia in children was demonstrated by several clinical studies, with the pre-myopic eyes showing more hyperopic peripheral refractive patterns [21]. Myopia regression (myopia increase after surgical emmetropization) has been described extensively after corneal refractive procedures. These refractive changes are not fully explained by changes in the corneal power. The crystalline lens as well as axial length have been implicated as potential causes of late myopia progression, particularly in higher myopes [22, 23]. Changing the peripheral focusing properties of the eye towards myopia with different optical methods proved to be effective in regulating myopia progression in children [24, 25]. It is expected that the peripheral myopic defocus induced by surgical corneal reshaping to correct myopia might act as a stimulus for lower axial elongation, according to the theories of refractive error development.

It could be argued that the mechanisms that induce axial growth control later in life are not the same as in children where myopia control with optical methods has shown efficacy. Additionally, peripheral defocus induced by surgical reshaping treatments is weaker than that induced by orthokeratology [17]. In any case, we aim to describe in further detail the changes induced by both treatments when analyzed in a more comprehensive way compared with previous studies that evaluated only the horizontal meridian. Therefore, the goal of the present study was obtaining further information on the optical properties of the post-LASIK cornea, which might help us better understand post-surgical growth regulation in adults who previously undergone corneal reshaping procedures. To our knowledge, this is the first study evaluating the relative peripheral refraction in OK and LASIK patients across different orientations of the visual field (horizontal, vertical and oblique meridians).

## Methods

### Subjects and inclusion criteria

In this prospective study, patients undergoing LASIK surgery or OK to correct low-to-moderate myopia were evaluated before and after 3 months of LASIK treatment (mean ± SD: 124.3 ± 12.8 days) and 1 month of OK treatment (mean ± SD: 37.0 ± 3.0 days). The protocol for contact lens fitting and the surgical protocol have been previously described [9, 17].

The inclusion criteria required the absence of any eye disease or injury and not taking ocular or systemic medication. Subjects should have a stable refractive error within the last 2 years to be considered for surgery. Before any treatment (OK lens adaptation or surgery), a complete optometric and ophthalmological examination was performed. After both treatments, the results of all patients were satisfactory with respect to residual refractive error (spherical equivalent within ±0.50 D), visual acuity (at least 20/20 visual acuity under high contrast and photopic conditions at 6 m), regularity and centering of the treatment zone.

The study followed the tenets of the Declaration of Helsinki and was approved by the School of Science at the University of Minho, Braga, Portugal. Measurements were obtained from 26 eyes of 26 subjects undergoing non-customized LASIK at the ophthalmological clinic Novovision (Madrid, Spain) and 34 right eyes of university students adapted with OK contact lenses to treat myopia between − 0.75 and − 5.25 D (M) (cylinder <− 1.00 D). A consent form was signed by each patient after the procedures and the nature of the study were fully explained.

Monocular measurements of the subjective non-cycloplegic refraction were recorded. The endpoint of refraction was established by the criterion of maximum plus for best visual acuity. The intraocular pressure was checked before and after treatment with a non-contact tonometer [26].

### Central and off-axis refraction

Central and off-axis refraction were measured with the open-field Grand Seiko Auto-Refractometer/Keratometer WAM-5500 (Grand Seiko Co., Ltd., Hiroshima, Japan). The procedure followed in this experiment has been previously described [27, 28] for the 35° nasal (N) and 35° temporal (T) of horizontal visual field. In this experiment, 22 additional locations were added to obtain information on the vertical meridian (15° in the superior and inferior retina) and up to 20° of eccentricity in oblique directions (45–225° and 135–315°) in 5° steps.

Peripheral refraction measures were obtained under non-cycloplegic and non-pharmacological dilatation. It is unlikely that the accommodative effort to fixate the targets at 2.5 m will produce a significant change in the measurement of the peripheral refractive error profiles [29].

### Statistical analysis

The SPSS software package v.19 (SPSS Inc., Chicago, IL, USA) was used for statistical analysis. The peripheral refraction across different meridians will be represented as the change from pre-surgery to post-surgery profiles. Kolmogorov-Smirnov and Shapiro-Wilk tests were applied to evaluate the normality of the data distribution, for the OK and LASIK, respectively. Paired samples *t*-test or Wilcoxon Signed Ranks Test were used for paired comparisons between baseline and post-treatment values, depending on the normality of the data. Statistical significance was considered when the *p* value was less than 0.05. Changes in relative peripheral refraction across vertical, horizontal and two oblique meridians (3D) after both treatments and the locations across the visual field showing statistically significant changes were identified. Peripheral myopization or peripheral myopic defocus refers to a change towards more myopic spherical equivalent refraction in post-treatment compared to pre-treatment measurements.

## Results

### Orthokeratology

Thirty-four right eyes of university students with a mean age of 25.2 ± 3.7 years (20 to 41 years, 13 females − 38.2% – and 21 males) constituted the OK group. Average pretreatment spherical equivalent was − 1.95 ± 1.27 D ranging from − 0.88 to − 5.25 D (cylinder − 0.33 ± 0.37 D).

**Fig. 1:**
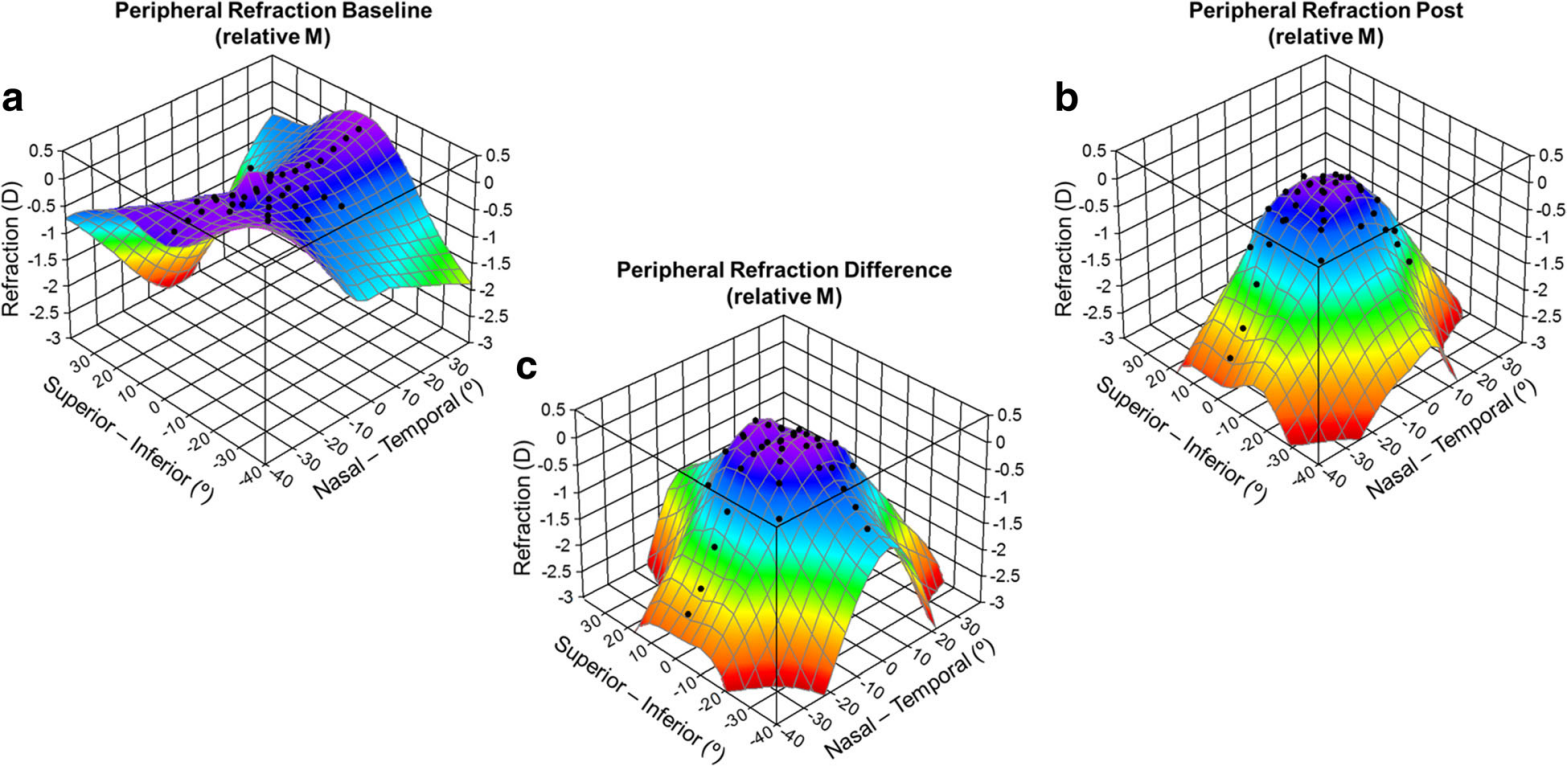
Three-dimensional representation of axial and peripheral RPRE (relative M) across horizontal (70° central in 5° steps), vertical (30° central in 5° steps) and two oblique (40° central in 5° steps) meridians in myopic healthy eyes before (**a**) and after (**b**) OK treatment and respective differences (**c**)

**Fig. 2:**
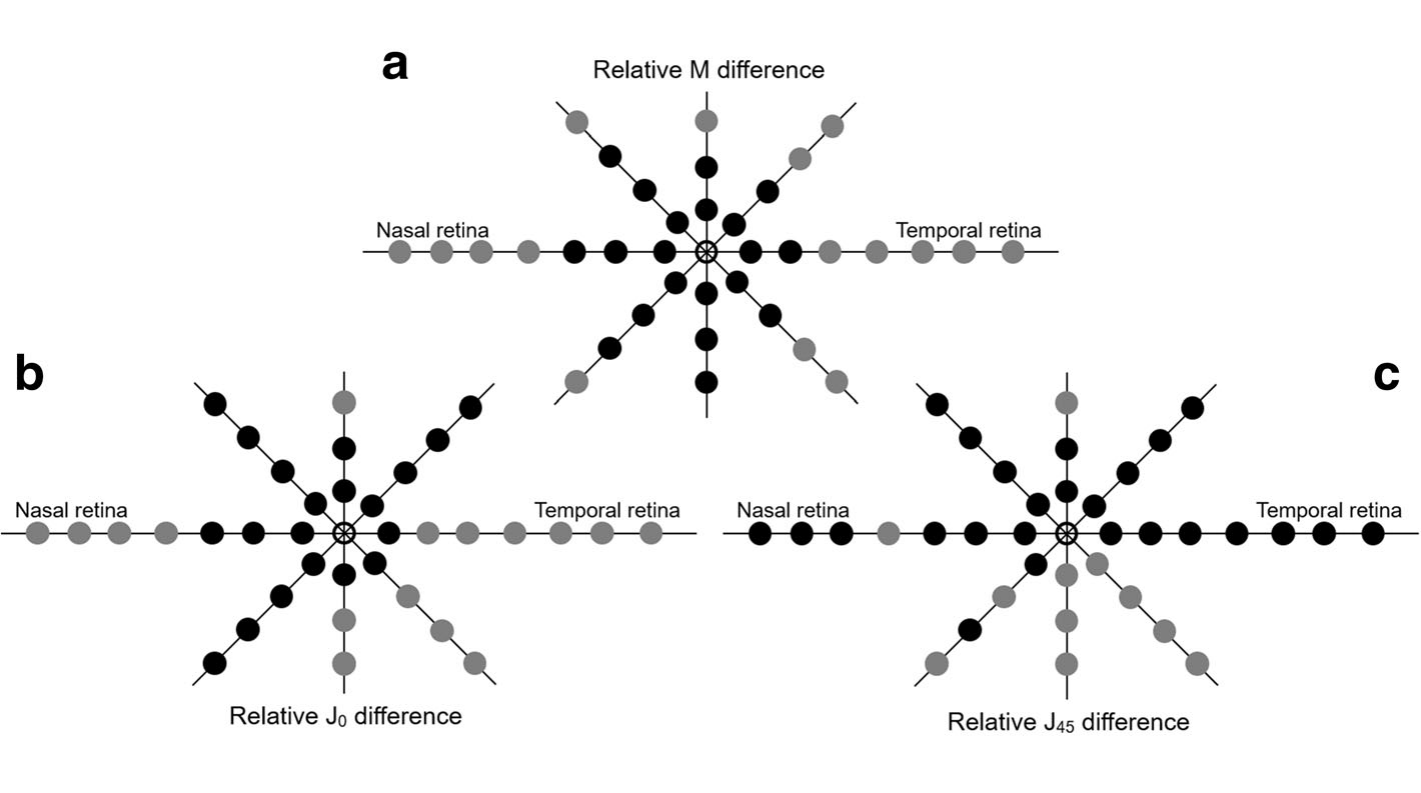
Schematic representation of statistical significance of changes induced by orthokeratology (OK) at different retinal eccentric locations represented by grey dots when comparing post-treatment against pre-treatment RPRE for M (**a**), J0 (**b**) and J45 (**c**)

Figure 1 shows a 3D representation of the relative peripheral refraction in the OK group (as M). The peripheral refractive errors prior to orthokeratology treatment suffered a small change; the maximum difference between the central and peripheral refractive error was 0.82 D more myope at 15° superior (Fig. 1a). However, post-treatment results showed an increase of myopic relative refraction at several eccentric locations shown in Fig. 1b. M differences between pre- and post-treatment were represented schematically in Fig. 1c. From Fig. 2a, the differences in the vertical meridian were statistically significant (*p* < 0.001, grey) only in the 15° superior location. Across the horizontal meridian, significant differences were found above 20°N (including) and above 15°T (including). In oblique directions, the significant differences were found at more eccentric locations of 20° at all meridians and at 15° on the temporal side. Greater M differences were observed at the horizontal meridian: − 2.30 ± 1.79 D (35°N) and − 2.54 ± 1.32 D (35°T). The differences at central 30° locations were very small and without statistical relevance (differences inferior to 0.46D – Fig. 2a). J0 and J45 differences are represented at Fig. 2 (b and c, respectively).

### LASIK

Twenty-six right eyes of subjects with mean age of 30.4 ± 4.8 years (20 to 37 years, 11 females − 42.3% – and 15 males) were included in the LASIK group. The average preoperative spherical equivalent was − 2.12 ± 0.92 D (from − 0.75 to − 3.88 D, cylinder − 0.52 ± 0.27 D).

**Fig. 3:**
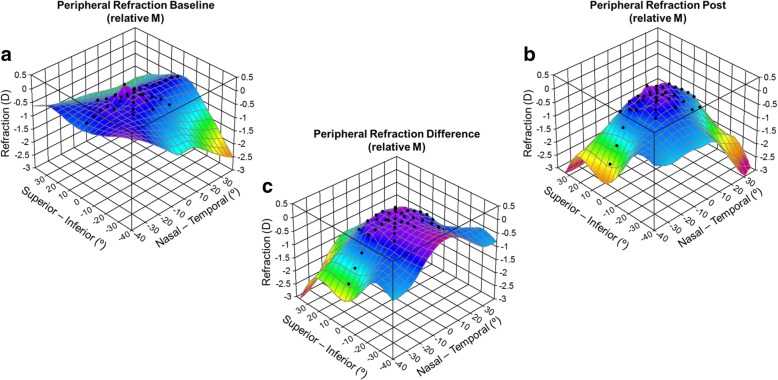
Three-dimensional representation of axial and peripheral RPRE (relative M) across horizontal (70° central in 5° steps), vertical (30° central in 5° steps) and two oblique (40° central in 5° steps) meridians in myopic healthy eyes before (**a**) and after (**b**) LASIK surgery and respective differences (**c**)

Figure 3 illustrates a 3D representation of the RPRE in the LASIK group. There was a small change in the peripheral refractive errors prior to treatment with LASIK; the maximum difference between the central and a peripheral refractive error is 0.64 D more myope at 20° superior (Fig. 3a).

**Fig. 4:**
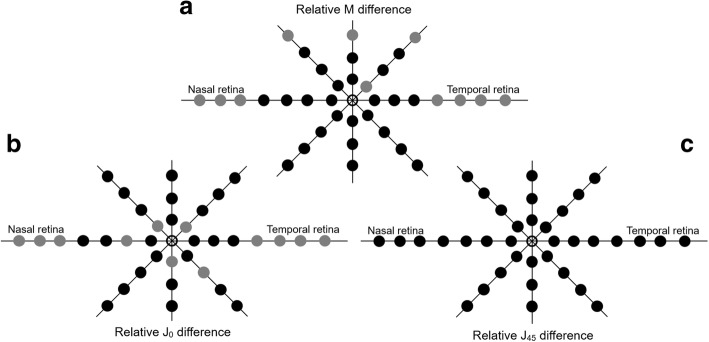
Schematic representation of statistical significance of changes induced by laser surgery (LASIK) at different retinal eccentric locations represented by grey dots when comparing post-treatment against pre-treatment RPRE for M (**a**), J0 (**b**) and J45 (**c**)

There were no differences in RPRE (M) values between before and after LASIK surgery over the central 30° across the horizontal meridian and at 20°N, as shown in Fig. 3c. Statistically significant differences were found in the vertical orientation at 20° superior (diff = − 0.24 ± 0.29 D, *p* = 0.001). Across oblique meridians, we found statistically significant differences in the M component in the superior hemifield (retina superior temporal/superior nasal). Greater M differences obtained at horizontal retina were of − 1.52 ± 1.06 D and − 1.17 ± 0.97 D at 35° nasal and temporal, respectively; remaining differences were inferior to 1 D and inferior to 0.25 D at central 40°. J0 (Fig. 4b) and M (Fig. 4a) values did not reveal differences at central 30° (horizontal meridian) except at 10°N (diff = − 0.12 ± 0.26 D, *p* < 0.01). Significant differences for the remaining vertical and oblique meridians for J0 and J45 values can be perused in Fig. 4b and c, respectively.

**Fig. 5:**
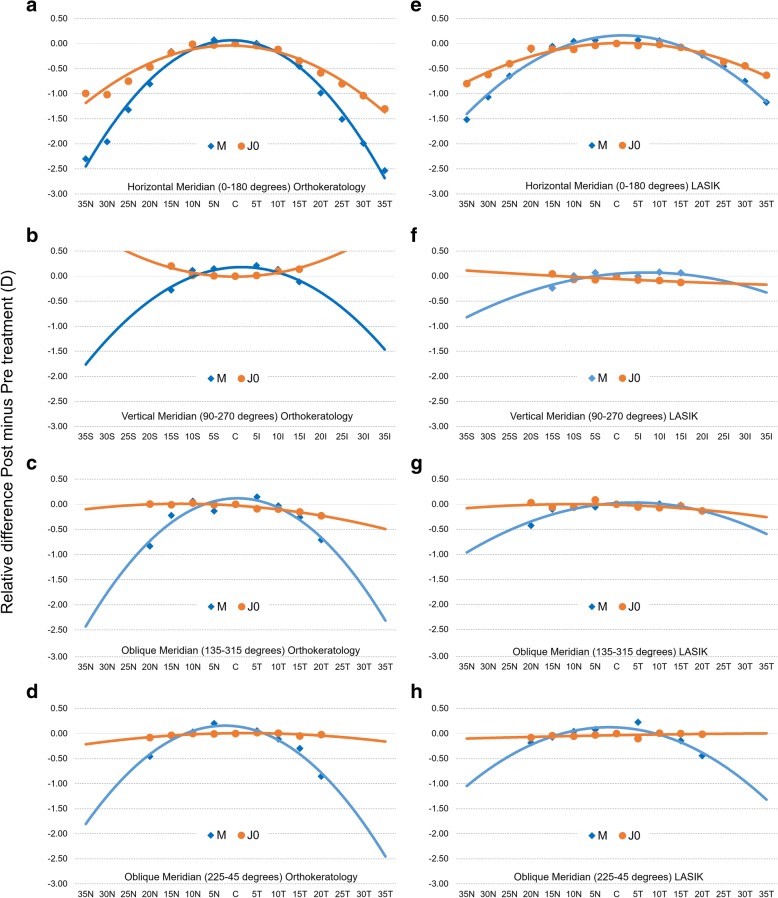
Two-dimensional representation of axial and peripheral RPRE (relative M and J0 for post minus pretreatment) across horizontal (70° central in 5° steps [**a**, **e**]), vertical (30° central in 5° steps [**b**, **f**]) and two oblique meridians (40° central in 5° steps [**c**, **g** and **d**, **h**]) for OK and LASIK surgery, respectively

Figure 5 shows a 2D representation of the RPRE (post minus pretreatment) in the OK (a to d) and LASIK (e to h) groups, for the four meridians in the components M and J0. These graphs represent the expected change in the Rx profile for any eye undergoing the same treatment as long as it has a similar myopia magnitude before the treatment.

## Discussion

We observed a general trend towards changing the peripheral refraction, but this was only statistically significant in 11 (LASIK) and 16 (OK) points of the 36 locations measured, as shown in Figs. 2 and 4. As described in the literature, these peripheral changes are obtained at the cost of spherical aberration induced by central ablation for myopic treatments [19, 30, 31]. This is the first study addressing the clinical measurement of the peripheral refraction before and after LASIK treatments at different meridional eccentricities [17]. Despite the negative implications in the optical quality of the eye, these outcomes could be beneficial for preventing myopia progression. Ma et al. showed that the pattern of peripheral refraction change towards a more myopic profile beyond the central 20° of eccentricity after myopic LASIK procedures [18]. In this pioneering work, they showed that the changes in the peripheral refractive profile rendered by myopic LASIK surgery were similar to those recently observed with OK. To some degree, this effect is thought to contribute to the reduction of myopia progression [8, 9, 32]. A similar effect could be observed in patients fitted with dominant design multifocal contact lenses that induce relative peripheral myopia [33, 34], which could be related to the recently demonstrated myopia retention [32].

The results of the present study show that LASIK induces a change in the relative peripheral refractive error, producing a myopic peripheral visual field after the procedure with no significance in the temporal visual field. Within the optic zone created by the laser, the central visual field became emmetropic. Furthermore, relative peripheral myopization occurs at all meridians studied. Nevertheless, the more significant effect was observed in the horizontal direction of the visual field where more eccentric locations were evaluated. These findings are related with previous results of central and peripheral (horizontal) post-surgery corneal curvature [11].

Compared to LASIK, OK treatment induces significant changes in the peripheral refractive pattern for a larger field area. While OK induces significant changes beyond the central 15°–20°, LASIK induces such changes beyond 20°–25°. This is related with the smoother transition of curvature between central treated and peripheral non-treated cornea [11]. Previous studies demonstrated that the baseline myopia in OK and LASIK patients is strongly correlated with the change towards peripheral relative myopization [9, 17]. Despite the slightly higher values of the average baseline myopia in the LASIK group, we observed that the peripheral relative myopia after the surgery was lower than in the OK group. This agrees with previous findings reporting a lower front corneal surface steepening at the edge of the treatment zone after LASIK compared to OK [11]. In OK, the greater increase in paracentral corneal power derives from the redistribution of the tissue, contrary to LASIK ablation of the central tissue [11]. In LASIK, the relative peripheral myopia is decreased by a larger optical zone and smoother transition areas [17].

There is controversy surrounding the use of refractive surgery in children [35, 36]. Several studies reported that surgical procedures such as radial keratotomy and laser-assisted procedures, may prevent refractive and anisometropic amblyopia in younger patients [37–41]. It was not the intention of this study to advocate for the use of corneal refractive procedures in children with the purpose of myopia control. Instead, the present results provide new information to better understand the reported late myopic regression in adults undergoing corneal refractive procedures for myopia correction. The investigation of this specific topic would further require a controlled clinical trial to evaluate and compare the eventual axial elongation of the eye in myopes undergoing surgery (regression) against the growth exhibited by non-surgical patients (progression).

Mathur et al. studied the optical quality after OK [10] and LASIK [20]. They performed measurements across the 42° horizontal vs 32° vertical central visual field in samples of 3 (OK) and 6 (LASIK) subjects and observed an increase of high-order aberration at peripheral locations after both treatments. Ehsaei et al. [42] studied refractive error across four meridians in healthy myopic subjects and emmetropes and they obtained comparable results with eccentricity-dependent profile, to the overall eccentricities studied, such as shown by Shen et al. [43]. Most of the previous studies with respect to the refractive profile before and after refractive treatments only reported results from the horizontal meridian [9, 17] or horizontal and vertical meridians [10, 20, 32]. Autorefractometers were mostly used [9–11, 17], but wavefront sensors [10, 20] were also introduced in this kind of evaluation since there was a good correlation between autorefractometers and Hartmann-Shack aberrometer values [44]. A new device capable of measuring from − 50° to + 50° in 10° steps in less than half a second was introduced [45]. This device allows the measurement of refractive error and ocular aberrations along horizontal, vertical, and five oblique meridians (i.e., 15°, 30°, 45°, 60°, and 75°) of the visual field for far and near distances.

The present study represents an overview of RPRE after refractive corneal reshaping treatments (OK and LASIK) across vertical, horizontal and two oblique meridians using a commercially available open-field autorefractor. In future studies, horizontal, vertical and oblique directions should be measured to better understand the visual experience across the visual field in the context of myopia progression or myopia regression studies.

Although this is a non-intentional side effect in the context of corneal refractive surgery, the changes in peripheral defocus after surgery might play some role in the late axial elongation, which myopic patients could potentially experience. Future longitudinal randomized and controlled trials are needed to evaluate the possible effect of manipulation of corneal optics in the context of refractive surgery in late myopia regression/progression in adults.

## Conclusion

Orthokeratology changes the relative peripheral refractive error in the 360 degrees of the visual field and this information might help to understand the overall effect of myopia control observed in other studies with this treatment option.
